# Detection of seizure patterns with multichannel amplitude-integrated EEG and the color density spectral array in the adult neurology intensive care unit

**DOI:** 10.1097/MD.0000000000012514

**Published:** 2018-09-21

**Authors:** Ji Sun, Dihui Ma, Yudan Lv

**Affiliations:** a Department of Pediatric Neurology; b Department of Neurology and Neuroscience Center, The First hospital of Jilin University, ChangChun, P. R. China.

**Keywords:** aEEG, CDSA, false-negative, false-positive, sensitivity

## Abstract

This study's purpose was to determine the sensitivity, false-positive and false-negative of seizure detection in adult intensive care by amplitude-integrated electroencephalography (aEEG) and color density spectral array (CDSA).

30 continuous electroencephalogram (EEG) recordings were randomly performed in 3 digital EEG-recording machines, 3 specialized neurophysiologists participated in this study, underwent 4 hours of training of CDSA and aEEG, marked any epochs suspected to be seizures without access to the raw EEG. The results will be compared and analyzed with continuous EEG reading to consider sensitivity, positive or negative rate.

The recordings in this study, comprised 720 hours of EEG containing a total of 435 seizures. The median sensitivity for seizure identification was 80% of CDSA and 81.3% of aEEG, Median false-positive was 4 per 24 hours of CDSA, and 2 per 24 hours of aEEG display, Median false-negative was 4 per 24 hours of CDSA, and 4 per 24 hours of aEEG display. The time spent in identification of seizures by CDSA and aEEG was much time-saving than continuous EEG-reading.

In this study, both CDSA and aEEG have a higher sensitivity but lower false-positive or missed rate in the interpretation of seizure identification in adult NICU.

## 1 Introduction

Patients in Neurology Intensive Care Unit (NICU) almost lost consciousness that was mainly caused by stroke, intracranial infection, status epilepticus or others. The seizures or subclinical seizures have occurred in 16% to 48% of critically brain-injured patients,^[1,2]^ which may bring unexpected consequence. When there is a clinical suspicion of seizures, a routine electroencephalogram (EEG) is administered, and proper treatment in time may reduce the damage to brain. However, the majority of electrographic seizures are subclinical.^[3–5]^ Therefore, continuous EEG monitoring is required for prompt and reliable electrographic seizure detection.

Although continuous electroencephalography (cEEG) is the standard method to confirm epilepsy seizures, unfortunately, this tool has its limitations, such as, in most units, equipment, technicians, and experienced clinical neurophysiologists are not available 24 hours per day. Besides this, the majority of electrographic seizures are subclinical epileptiform discharges.^[6]^

So, amplitude-integrated electroencephalogra*p*hy (aEEG) and color density spectral array (CDSA) have become available tools that are now used routinely in many ICU or NICU. aEEG depicts time-compressed and rectified EEG amplitude on a semi-logarithmic scale, and is now commonly employed to monitor cerebral function.^[7,8]^ CDSA applies fast-Fourier transformation (FFT) to convert raw EEG into a time-compressed and color-coded display, which also has been termed as color spectrogram.^[8–10]^ Clinical applications of CDSA have included identifying seizures,^[8]^ identifying subclinical seizures,^[8]^ monitoring cerebral function, identifying cerebral ischemia,^[11]^ monitoring depth of sedation.^[12]^ While aEEG and CDSA provided many utilities for clinical application, also remained controversial debate.^[13–15]^ Nowadays, there have been few reports on the use of aEEG and CDSA for seizure identification in adult NICU.^[16,17]^ This was the reason why we performed this trial to investigate whether clinical and subclinical seizures can be detected with aEEG and CDSA frequently and accurately.

## 2 Materials and methods

### 2.1 Clinical materials

We conducted a randomized, controlled trial, all continuous EEG recordings were performed in 30 patients aged 17 years to 52 years in our epilepsy center between June 2011 and October 2012. The Research Ethics Board of the First Hospital of Jilin University approved this study, written informed consent was obtained from parents or guardians before randomization.

### 2.2 Entry and exclusion criteria

All continuous EEG recordings included at least 24 hours’ duration that were performed for the following indications: Encephalitis with frequent seizures, Convulsive status epilepticus, Nonconvulsive status epilepticus, Paroxysmal symptom suspected to seizures, Subclinical electrographic-seizure.

We excluded EEG recordings containing more than 20 seizures per hour and recordings whose voltage of ictal evolution under 20uv, because these would haven’t been identified or displayed by CDSA and aEEG appropriately. EEG recordings containing less than 5 seizures per 24-hours were also excluded because false-positive or false-negative rate might not be acceptably calculated for EEGs with low rates of seizures.

### 2.3 Study design

30 continuous EEG recordings were randomly performed in 3 digital EEG- recording machines: 20 recordings containing seizures and 10 control files without seizures, each digital EEG-recording machine has 10 recordings. Three neurophysiologists participated in this study. Both Reviewer 1 and Reviewer 2 were board-certified, with at least 3 years of continuous EEG reading experience, Reviewer 3 only has 1 year of reading experience, but none had any prior experience using CDSA or aEEG. At first, each participant was presented with 1 digital EEG recording machine at random, then took turns to complete all 30 recording-reading on 3 machines respectively. Participants first underwent 3 hours of training, during which they were introduced to the theoretical basis of CDSA and aEEG, followed by extensive hands-on training on the recognition of seizures and various artifacts. During testing, each participant is not allowed to consult with each other. Participants tried to mark any epochs that has been suspected to be seizures on CDSA and aEEG displays, after testing, which will be compared and analyzed with continuous EEG to consider the sensitivity, positive or negative rate. The sensitivity for seizure identification, the false-positive rate or false-negative rate per 24 hours was calculated by comparing the suspected seizures marked on CDSA and aEEG displays to the raw EEG, which has been considered as golden standard.

### 2.4 aEEG and CDSA recording and analysis

The aEEG and CDSA were recorded with the cerebral function monitor (Graphic version, Biology Inc, America). 6 channels EEG were derived from bilateral electrodes according to the international 10 to 20 system (F3-C3,C3-P3,P3-O1;F4-C4,C4-P4,P4-O2). The filtered signal was rectified at 0.1 Hz and 70 Hz. Continuous EEG recordings were transformed to CDSA and aEEG by using the quantitative EEG tools built into the Olympic EEG reviewing software (Natus Systems Inc., Biologic, America). The seizures identified by CDSA and aEEG were compared and analyzed with continuous EEG reading. Electrographic seizures were identified using published criteria.^[6]^ Regarding to all ambiguous cases during continuous EEG reading, 3 participants consulted, and a consensus was reached. Then, identification, comparison, analysis, and calculation were made.

### 2.5 Statistics analysis

Statistical analysis was performed by using SPSS 15.0 (SPSS Inc, Chicago, IL). Comparisons of false-negative rate between aEEG and CDSA were made with *X*^*2*^ tests, A value of *P* < .05 was considered significant. Fleiss-*K* for multiple raters was calculated to determine the level of interrater agreement above chance (*K*, 0.41–0.60 Moderate agreement; 0.61 to 0.80 Substantial agreement; 0.81 to 1.00 Almost perfect agreement).

## 3 Results

### 3.1 The characteristics of the 30 continuous EEG recordings

Thirty recordings contained 20 seizure-recordings and 10 control recordings without seizures. The recordings comprised 720 hours of EEG containing a total of 435 seizures. The common indications for continuous EEG monitoring were Convulsive status epilepticus, suspected Nonconvulsive status epilepticus, Paroxysmal symptom suspected to be seizures, Subclinical electrographic-seizure., Encephalitis, Epilepsy, Hypoxicischemic encephalopathy. Clinical characteristics are listed in Table 1.

**Table 1:**
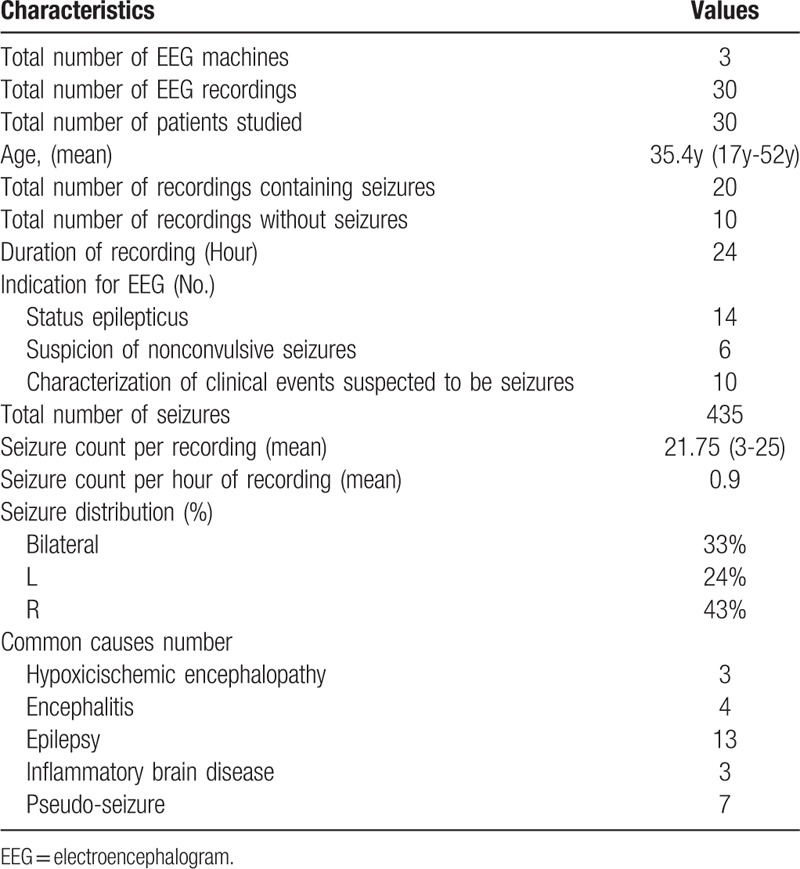
The characteristics of the 30 continuous EEG recordings.

### 3.2 Sensitivity

**Figure 1:**
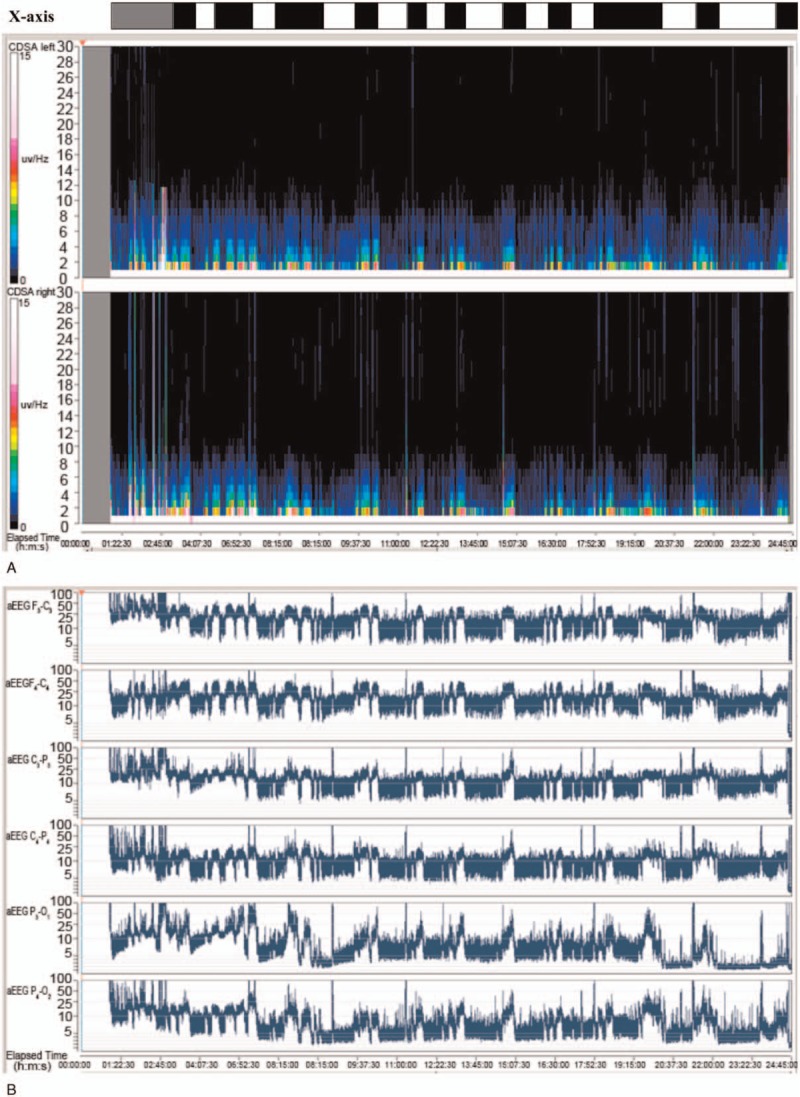
Displays of seizures identified by CDSA and aEEG. The CDSA displays of seizures (A) and aEEG displays of seizures (B) were compared to seizures identified by analyzing the raw EEG data (X-axis on top of the displays), which were concealed from the neurophysiologists during the actual testing. A cluster of seizures in the raw EEG data is indicated by a black box on the X-axis, while the body-movement artifact is indicated by a gray box on the X-axis. aEEG = amplitude-integrated electroencephalography, CDSA = color density spectral array, EEG = electroencephalogram.

The sensitivity for seizure identification per recording was calculated by comparing the suspected seizures marked by the neurophysiologists on CDSA and aEEG displays to the seizures identified by the gold standard of the raw EEG. When considering sensitivity across all EEG recordings, the 3 neurophysiologists correctly identified a median of 80% (range78.5%–84.1%) of seizures per recording using CDSA and a median of 81.3% (range 77.9%–83.9%) of seizures per recording using aEEG (Fig. 1). However, it was more variable of sensitivity among individual EEG recordings, the seizure identification rate (sensitivity) was 80% or greater among 11 recordings (55%) using CDSA; was 75% or greater and 80% less among 6 recordings (30%) using CDSA; was 70% greater and 75% less among 3 recordings (15%) using CDSA. The seizure identification rate was 80% or greater among 15 recordings (75%) using aEEG; was 75% or greater and 80% less among 5 recordings (25%) using aEEG; none was 75% less among recordings using aEEG. Overall, each EEG-recording has a sensitivity greater than 70% by both CDSA and aEEG. In our study, none of the 3 reviewers encountered at any recording in which they missed all seizures on CSDA, aEEG, or on both displays.

### 3.3 False-positive rate

False-positive rates were identified by 3 neurophysiologists across all EEG recordings. Overall, these median false-positive rates were quite low, corresponding to 4 false-positive per 24 hours of CDSA (2–4 n\24 h), and 2 false-positive per 24 hours of aEEG displayed (2–2 n\24 h). False seizures were 8 to 14 among the 20 files (40–70%) with seizures of CDSA and were 2 to 3 among the 10 files (20–30%) without seizures of CDSA. False seizures were 7 to 13 among the 20 files (35–65%) with seizures of aEEG, but were 2 to 3 among the 10 files (20–30%) without seizures of aEEG.

False-positive rate did vary among the 30 individual recordings: 3 EEG recordings (nos.7, nos.9 or nos.24) resulted in particularly high false-positive rates when interpreted by using CDSA for head-shaking artifact, body-movement artifact, which were misidentified as seizures by 3 reviewers.

### 3.4 False-negative rate

False-negative rate was calculated by the number of missed seizures that had not been marked per recording (24 h), on the basis of the golden standard of the raw EEG. Overall, median false-negative rates were low, corresponding to 4 missed rate per 24 hours of CDSA (3.5–4 n\24 h), and 4 missed rate per 24 hours of aEEG (3.5–4.5 n\24 h). Overall, 19.2% of seizures were completely missed when using CDSA and 19% of seizures were missed when using aEEG; there was no significant difference of false-negative rate between CDSA and aEEG (*P* > .05, *X*^*2*^). However, EEG recordings (nos.9, nos.15 or nos.18) resulted in high false-negative rates for following causes: low voltage, short duration, seizures that remained focal, or seizures that occurred in abundant discharges.^[8]^

### 3.5 Sensitivity, false-positive and false-negative rate between individual reviewers and interrater agreement

In this study, Sensitivity, false-positive, and false-negative rate did vary when interpreted by different individual reviewers (see in Table 2). Overall, the median sensitivity was 80% by using CDSA, and among 3 reviewers, the median sensitivity of reviewer 1 to 3 was respectively 80% (75–86.9%), 78.5% (70.6–84.2%), 84.1% (70.6–95%); the median sensitivity was 81.3% by using aEEG, and among 3 reviewers, the median sensitivity of reviewer 1 to 3 was respectively 81.3% (75–86.4%), 77.9% (72.7–83.9%), 83.9% (62.5–90%). The median false-positive rate was 4 n\24 h by using CDSA, and among 3 reviewers, the median value of reviewer 1 to 3 was respectively 4 (2–6), 4 (1–8), 2 (1–6); the median false-positive rate was 2 n\24 h by using aEEG, and among 3 reviewers, the median value of reviewer 1 to 3 was respectively 2 (1–5), 2 (1–4), 2 (1–4). The median false-negative rate was 4 n\24 h by using CDSA, and among 3 reviewers, the median value of reviewer 1 to 3 was respectively 4 (3–8), 4 (3–9), 3.5 (1–6); the median false-negative rate was 4 n\24 h by using aEEG, and among 3 reviewers, the median value of reviewer 1 to 3 was 4 (3–6), 4.5 (3–7), 3.5 (2–6) respectively.

**Table 2:**
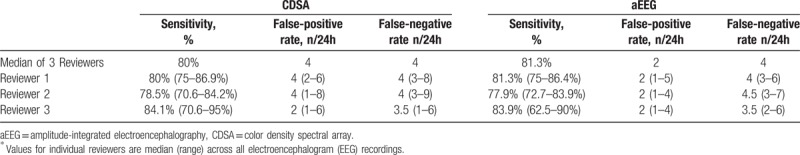
Comparisions of sensitivity, false-positive and false-negative rate between individual reviewers using CDSA^∗^ and aEEG^∗^.

κscores, a measure of interrater agreement above that indicated moderate agreement among all neurophysiologists when using CDSA (κ=0.52) and substantial agreement (κ=0.68) when using aEEG to identify seizures.

### 3.6 Time spent in training and identification of seizures by CDSA and aEEG between individual Reviewers

In this study, both 2 neurophysiologists were board-certified, with 3 years of continuous EEG reading experience and 1 has 1 year of reading experience, but none had any prior experience using CDSA or aEEG. Participants firstly underwent 3 hours of training, during which they were introduced to the theoretical basis of CDSA and aEEG, followed by extensive hands-on training on the recognition of seizures and various artifacts, and at last, 3 participants have spent 1 hour in passing the qualified exams. In total, 3 neurophysiologists only spend 4 hours in training and educating of CDSA and aEEG.

In this study, Reviewer 1 spent median time of 33.4 minutes in identification of seizures by CDSA and 32.7 minutes by aEEG; Reviewer 2 spent median time of 30.9 minutes in identification by CDSA and 30.5 minutes by aEEG; Reviewer 3 spent median time of 55 min in identification by CDSA and 48.5 minutes by aEEG. However, in previous, Neurophysiologists almost spent 1 half or 2 hours on continuous 24 hours-EEG-reading, which was much more time-consuming than the using of CDSA and aEEG.

## 4 Discussion

Epilepsy seizure is an important and common disease in the NICU at high risk. The practical difficulties in obtaining expeditious continuous EEG recordings in many NICUs have led to the use of aEEG as an adjunct for electrographic seizure detection. In previous studies, Abend et al demonstrated a sensitivity of 88% for identifying long seizures, 40% for brief seizures, and 20% for slowly evolving seizures, and a false-positive rate of 0 to 2 per hour, by using quantitative EEG trend of 6-channel to display.^[18]^ Stewart et al used 8-channel CDSA and aEEG to display, invited 3 neurophysiologists to identify, and made a median sensitivity, 83.3% of CDSA and 81.5% of aEEG.^[8]^ In neonates, Rennie et al have evaluated 1- or 2-channel aEEG displays, and reported a wide range of sensitivities for seizure identification from 26% to 76%.^[15,19]^

However, in this study, we used a 6-channel envelope trend to interpret. There was a median sensitivity of 80% per recording using CDSA and a median sensitivity of 81.3% per recording using aEEG, which was somewhat different from previous studies, there may be some factors such as: levels of expertise in CDSA and aEEG interpretation; the number of channels; the amplitude, the duration of ictal-EEG evolution; the number of seizures per hour. In this study, the 3 neurophysiologists only accepted 4 hours of training and without access to the raw EEG, some recordings have low amplitude of ictal-EEG, which may result in lower sensitivity than Abend and Stewart, however, more channels for CDSA and aEEG display may result in higher sensitivity than Rennie.

Just like previous studies, false-positive rate across all recordings marked by 3 reviewers in this study was very low, 4 false-positive per 24 hours of CDSA, and 2 false-positive per 24 hours of aEEG. False-positive rates were sometimes associated with artifacts, such as movement, electromyography (EMG) or electrodes.^[16,20]^ false-positive rates did vary among the 30 individual recordings, Nos.9 or Nos.24 resulted in particularly high false-positive rates when using CDSA and aEEG, for head-shaking artifact, while far less frequent in the other recordings.

Besides, false-negative rate in this study was quite low, corresponding to 4 missed rate per 24 hours of CDSA, and 4 missed rate per 24 hours of aEEG displayed, there was no significant difference of false-negative rate between CDSA and aEEG (*P* > .05, *X*^*2*^). False-negative rate in our study was associated with low amplitude and short duration, such as Nos.6 has a low amplitude (<35 UV) and Nos.12 has a short duration (<20 s). aEEG is at least amplitude-based and CDSA is time-compressed-based, which may be limitations to false-negative rate. Difficulty in detecting short seizures is a known limitation of CDSA, due to time compression of the display.^[19]^ Difficulty in detecting low amplitudes is a known limitation of aEEG, due to amplitude-based display.^[19,21]^ Otherwise, focal seizures, context of abundant interictal epileptiform discharges or high-amplitude, generalized background have been reported to account for the poor detectability.^[8]^

In this study, sensitivity, false-positive and false-negative rate of seizure identification utilized by aEEG and CDSA has been reported, which have been few descriptive before, especially in adult NICUs, which may be important for widely application of aEEG and CDSA in future. However, there were some limitations in this study: participants have only taken 4 hours to pass the qualified- examination and interpret the EEG-recordings by CDSA and aEEG with poor experience, which may result in lower sensitivity, but higher false-positive and false-negative rate. Besides this, in our study, we excluded EEG recordings containing more than 20 seizures per hour, because “seizures” were likely to be confused with the artifacts after cEEG were transformed to CDSA or aEEG in this case. We also excluded voltage of ictal evolution under 20uv, because in this range, transformed EEG-recordings may not be displayed perfectly due to the low voltage. The reason for us to exclude EEG recordings containing less than 5 seizures per 24-hours is that sensitivity, false-positive and false-negative rate may not be interpreted by CDSA and aEEG more credibly due to the small numbers of sample size. So the exclusion criteria may be detrimental for EEGs with lower rates of seizures or higher rates of seizures. As far as the artifact, it may be misinterpreted when interpreted by CDSA and aEEG, either be neglected or mistaken as an “event”, combining with continuous EEG might solve this problem, however, in our study, participants have no opportunity to approach the raw EEG data before test, in order to explore the sensitivity, false-positive and false-negative rate by aEEG and CDSA exclusively, if these results can be acceptable, it will be much more credible when combining with the continuous EEG. Furthermore, the accuracy of CDSA and aEEG in this study may be a little different from previous studies for different channels number, different samples and different patient population, which may be improved by changing recording technique, increasing samples or applying other quantitative EEG modalities in future research.

## 5 Conclusion

Our findings demonstrate that CDSA and aEEG have a high sensitivity and low false-positive and false-negative rate in interpretation of seizure identification in adult NICU, which will be useful and time-saving tools for raw screening, and will be more credible to combine with continuous EEG to minimize the false-positive and false-negative rate and maximize the sensitivity, but won’t replace careful review of continuous EEG data. However, whether these quantitative electroencephalogram (QEEG) display tools are suitable for widespread application in NICU, ICU, or other places requires further study.

## Author contributions

Dihui Ma and Yudan Lv are both corresponding authors of this paper.

**Conceptualization:** Yudan Lv.

**Data curation:** Yudan Lv.

**Formal analysis:** Ji Sun, Yudan Lv.

**Investigation:** Yudan Lv.

**Methodology:** Ji Sun, Yudan Lv.

**Project administration:** Dihui Ma.

**Resources:** Dihui Ma, Yudan Lv.

**Software:** Yudan Lv.

**Supervision:** Dihui Ma.

**Validation:** Dihui Ma, Yudan Lv.

**Visualization:** Dihui Ma.

**Writing – original draft:** Ji Sun, Yudan Lv.

**Writing – review & editing:** Ji Sun, Dihui Ma, Yudan Lv.
